# Fetal Health Classification from Cardiotocograph for Both Stages of Labor—A Soft-Computing-Based Approach

**DOI:** 10.3390/diagnostics13050858

**Published:** 2023-02-23

**Authors:** Sahana Das, Himadri Mukherjee, Kaushik Roy, Chanchal Kumar Saha

**Affiliations:** 1 School of Computer Science, Swami Vivekananda University, Kolkata 700121, India; 2 Department of Computer Science, West Bengal State University, Kolkata 700126, India; 3 Department of Obstetrics and Gynecology, Biraj Mohini Matrisadan and Hospital, Kolkata 700122, India

**Keywords:** cardiotocograph, fetal heart rate, random forest, sensitivity, specificity, SVM

## Abstract

To date, cardiotocography (CTG) is the only non-invasive and cost-effective tool available for continuous monitoring of the fetal health. In spite of a marked growth in the automation of the CTG analysis, it still remains a challenging signal processing task. Complex and dynamic patterns of fetal heart are poorly interpreted. Particularly, the precise interpretation of the suspected cases is fairly low by both visual and automated methods. Also, the first and second stage of labor produce very different fetal heart rate (FHR) dynamics. Thus, a robust classification model takes both stages into consideration separately. In this work, the authors propose a machine-learning-based model, which was applied separately to both the stages of labor, using standard classifiers such as SVM, random forest (RF), multi-layer perceptron (MLP), and bagging to classify the CTG. The outcome was validated using the model performance measure, combined performance measure, and the ROC-AUC. Though AUC-ROC was sufficiently high for all the classifiers, the other parameters established a better performance by SVM and RF. For suspicious cases the accuracies of SVM and RF were 97.4% and 98%, respectively, whereas sensitivity was 96.4% and specificity was 98% approximately. In the second stage of labor the accuracies were 90.6% and 89.3% for SVM and RF, respectively. Limits of agreement for 95% between the manual annotation and the outcome of SVM and RF were (−0.05 to 0.01) and (−0.03 to 0.02). Henceforth, the proposed classification model is efficient and can be integrated into the automated decision support system.

## 1. Introduction

The perinatal mortality rate is defined as the total number of stillbirths and deaths that take place during the first seven days of life per 1000 live births. According to UNICEF, in low- and middle-income countries this figure stood at 19 per 1000 births by 2018, whereas for high-income and upper-middle-income countries this value was 3 and 7 per 1000, respectively South Asia and Sub-Saharan Africa have the highest perinatal mortality at 26% and 28%, respectively as reported by UNICEF [1]. Though in the beginning of 20th century the perinatal mortality in high-income countries was frighteningly high, it was drastically reduced with proper antenatal care, comprehensive indicators of C-section, and introduction of perinatal screening technologies such as cardiotocograph (CTG), ultrasound, fetal ECG, amnioscopy, and amniocentesis etc. [2].

It was found that the most common reasons for birth-related complications and perinatal deaths are prematurity, birth asphyxia, maternal hypertension, and septicemia [3]. Asphyxia arises due to prolonged deprivation of oxygen caused by the interruption in the placental blood flow that is caused by maternal pre-eclampsia, placental abruption, or umbilical cord prolapse. Inadequate delivery management causes the signs of asphyxia to be overlooked. Fetal heart rate during the intra-partum and ante-partum period gives indications of the signs of asphyxia that, if detected in time, can prevent irreversible organ damage or even death.

The main objective of intrapartum fetal monitoring is to recognize fetuses with risks of mortality and morbidity in order to ensure timely intervention. Cardiotocograph (CTG) is the most commonly used external monitoring system that continuously records the fetal heart rate (FHR) and uterine contraction (UC) to produce a visual display either electronically or on thermal paper. CTG has been in clinical practice for the last six decades and has enabled clinical practitioners to detect signs of fetal compromise at an early stage. However, studies have shown that continuous CTG monitoring of low-risk pregnancies led to an increase in unnecessary interventions and C-sections. To avoid this [4] international guidelines such as FIGO [5] NICE, NICHD [6] etc., have suggested the use of CTG only for high-risk pregnancies.

### 1.1. Physiology of Fetal Hypoxia

The autonomic nervous system (ANS), baroreceptors, and chemoreceptors control the pace of the fetal cardiac activity. The ANS consists of the sympathetic nervous system, which accelerates the heart rate, and the parasympathetic nervous system, which tries to reduce the heart rate. The baseline of the FHR is determined by the balance between these two nervous systems. When blood pressure increases, baroreceptors help reduce the heart rate, which in turn reduces blood pressure. This reduction in blood pressure prompts the baroreceptors to reduce the tone of the parasympathetic nerve and stimulate an increase in FHR and blood pressure. Chemoreceptors can sense the depletion of oxygen levels in fetal blood, which causes an increase the FHR to intensify the rate of oxygen input from the placenta. However, during hypoxemia, chemoreceptors cause the FHR to drop, resulting in an increase in blood pressure.

### 1.2. Problems of CTG Interpretation

Significant and methodical cardiotocography (CTG) interpretation is vital to minimize intrapartum fetal asphyxia and the related grim consequences. Nowadays, though there are widespread uses of CTG, it suffers from inter- and intra-observer variation which results in low specificity. To counter this problem Dawes et al. [7] introduced the first computerized version of CTG for automated feature extraction. A study that involved 469 subjects established that the fetal mortality rate when monitored by computerized CTG was four times less than the population whose CTG was interpreted visually. Despite the worldwide acceptance of CTG, the medico-legal issues have risen due to reasons such as improper interpretation of CTG and subsequent lack of timely action [8] Birth asphyxia-related litigations in the UK in the decade 2000–2010 totaled as high as GBP 3.1 billion. A total of 73.6% of US-based obstetricians faced litigations due to fetal neurological damage. These litigations not only affected the working lives of the clinicians but also had influenced them to opt for unnecessary C-sections as a safeguard. The lack of standard guidelines for interpretation and recognition of the FHR signals in the gray zone is another reason for misinterpretation. To address these issues, in recent times soft-computing-based methods have been explored to find better interpretation of FHR results, which yielded results that are comparable to the clinical interpretation. These systems are capable of distinguishing between normal and IUGR fetuses [9].

Automated interpretation of CTGs have not produced the desired result in predicting the pathological conditions. Soft-computing-based techniques were thus sought to identify the high-risk features with better accuracy. In fetal healthcare, machine learning had been used successfully to measure fetal weight [10] predict hypoxia, or estimate gestational age [11].

In this work, the authors propose a machine learning (ML)-based technique to classify the CTG by taking into consideration both the stages of labor separately. Standard ML algorithms such as MLP, SVM, random forest, and bagging were used for classification, and the most suitable classifier was selected using various statistical analysis metrics.

Arrangement of the paper is as follows: Literature study is elaborated in Section 2; Methodology is described in Section 3; Section 4 contains the Results and Discussion; and the Conclusion is given in Section 5.

## 2. Literature Study

Since the classical method for the interpretation and classification of CTG failed to produce convincing results, in last decade the focus of research had shifted towards the application of soft-computing-based techniques for the classification of CTGs. Comert et al. used the CTU-UHB dataset with 552 raw CTG data points to predict the fetal state with 87.9% accuracy [12]. In another work with the same dataset, they reported an accuracy of 91.8% and 93.4%, respectively, with an artificial neural network (ANN) and an extreme learning machine (ELM) Comert (2019) [13]. These models have shown high accuracy for normal and pathological states; however, the accuracy dropped to 59% for suspicious CTGs. Sundar et al. [14] generated a model using XGBoost with an accuracy of 96% for the pathological state but only 73% for suspicious state. Batra et al. [15] performed feature selection on the UCI machine learning repository’s CTG data.

Impulse response function, FHR baseline, and variability were used as inputs to SVM by Warrick et al. [16] to train the system to classify normal and pathological CTGs. A total of 50% of the pathological CTGs were correctly identified with FP = 7.5%. The training dataset contained 189 normal CTGs and 31 pathological ones. Dash et al. [17] exploited the dynamic nature of the FHR signal. They segmented the CTG record into shorter segments and extracted the features from each segment separately. The sequence of features from the segments were used as inputs to a Bayesian classifier. The true positive rate (TPR) and true negative rate (TNR) were 0.817 and 0.609, respectively, and thus outperformed SVM on the same dataset. The hidden patterns from the FHR were extracted by Chamidah et al. [18] (to classify the fetus as normal, irregular, or pathologic using k-means clustering with an accuracy of 90.64%.

Brocklehurst et al. [19] used numerical algorithms and ANN to effectively identify abnormal CTGs. Features such as signal quality, patient clinical data, and the major features of CTG were used as the input. Nuanes et al. [20] used uterine contractions, acceleration, and deceleration as input features to identify the rates of acidosis. In both the works the targets were labelled manually by the clinicians. Deceleration capacity was also used to predict acidemia with pH < 7.05 [9]. Another similar study was conducted with fractal analysis and the Hurst parameter as the input [21]. In these two studies, the AUCs were found to be 0.665 and 0.87, respectively. Dash et al. [17] used the standard parameters of CTG and applied generative models and Bayesian theory to predict acidemia; pH < 7.15 was used for providing the target labels.

Petrozziello et al. [22] used a multi-modal convolution neural network (MCNN) on 35000 CTG data points and had a false positive rate (FPR) of 15% and a TPR of 31%. Ogasawara et al. [23] used a database containing 162 normal and 162 abnormal CTGs to show that a deep neural network (DNN) performed better in classifying CTGs than either SVM or k-means clustering. Rahamayanti et al. [24] experimented with LSTM models that had 28, 14, and 42 nodes in their hidden layers. They used ReLU and Softsign activations with 32 batch size. The accuracies were 95–98% for different models.

Besides class imbalance, the major challenge in developing a robust soft-computing-based CTG interpretation model is class labeling. Many class labeling methods use weak metrics such as pH and healthy against HIE (hypoxic ischemic encephalopathy) without taking into consideration other CTG abnormalities. In this work, the authors have addressed this problem by annotating the CTG by different clinicians. Also, the classification of CTG without considering the progress in labor is not ideal because definition of normal features varies from first stage to second stage. The authors have taken this into account and performed the classification based on the stage of labor. The studies discussed above do not take into consideration the second stage of labor, which reduces the usefulness of the decision support system.

## 3. Methodology

**Figure 1:**
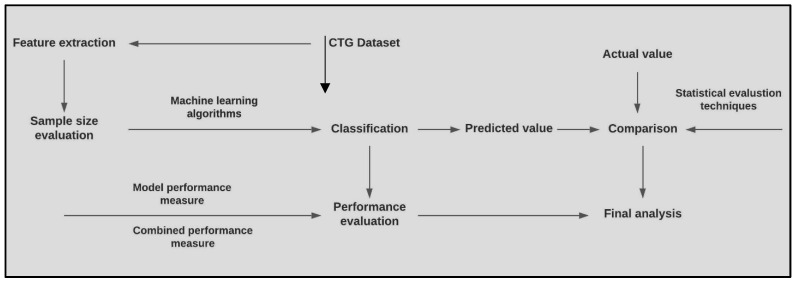
Overview of the proposed methodology.

Classification was performed separately on both the stages of labor. The duration of each stage of labor was roughly 30 min. Overview of the methodology is given in Figure 1.

### 3.1. Dataset Description

The Czech Technical University and University Hospital of Brno (CTU-UHB) database consists of 552 intrapartum records collected using the OB TraceVue System between 27th April and 6th August 2012. The duration of each record was at most 90 min. In the CTG records, the second stage of labor was not more than 30 min. From the 552 cases, only 46 were caesarean deliveries, whereas the remaining were vaginal deliveries. The following selection criteria had been taken into account [25]:

1. Mother’s age—plays a role in congenital disease. Women with age below 18 years were thus excluded from the study.
2. Weeks of gestation—affects the shape and behavior of the FHR signal. Hence, only fetuses with a gestational age greater than 36 weeks were included in the study.
3. Known fetal disease—a disease such as intrauterine growth retardation (IUGR) has an effect on FHR patterns. Fetuses with IUGR or other congenital defects were not part of the study.
4. Singleton pregnancies without complication were included.

Different labor outcome measures such as umbilical artery pH were also provided to facilitate objective classification. It is considered the most common outcome measure of fetal hypoxia. Besides pH, the following outcome measures were also provided in the database:

1. Base excess (BE) is a sign of metabolic hypoxia.
2. A base deficit in extracellular fluid (BDecf) is another measure of hypoxia.
3. Complete neonatal records of each baby. There was no neonatal morbidity, hypoxic-ischemic encephalopathy (HIE), or seizures.
4. Apgar score.

The following criteria had been applied to the CTG records:

1. Signal Length: Out of the 90 min of each CTG record, stage 1 was restricted to a maximum of 60 min. The minimum time length, $T_{d}$, needed was:
  (1) $$\{pH\leq 7.15;T_{d}\geq 30minspH>7.15;T_{d}\geq 40mins$$
  Time duration from the end of the first stage of labor to birth was less than 30 min. A maximum of 30 min of FHR was recorded for the second stage of labor to ensure the circumvention of adversarial outcomes that might occur at the end of this stage.
2. Missing signal: The quantity of missing signal was a maximum of 50% in the first stage of labor and was kept to an optimum in the second stage.
3. Noisy signal: Maternal heart rates were present in some of the recordings due to the inaccurate placement of the ultrasound probe.

The main parameters of the CTG signal were:

1. Length of stage 1 of the labor in minutes.
2. Length of stage 2 of the labor in minutes.
3. Time duration from the signal end to birth.
4. Percentage of noisy data in Window 1.
5. Percentage of noisy data in Window 1.
6. Overall percentage in Window 1.
7. Percentage of noisy data in Window 2.
8. Percentage of noisy data in Window 2.
9. Overall percentage in Window 2.

### 3.2. Extraction of the Features

The main features of CTG had been extracted in some previous works by the author. The authors considered two frequency domain parameters, i.e., baseline variability and sinusoidal heart rate pattern (SHR) and three time domain parameters, i.e., FHR baseline, acceleration, and deceleration. A summary of the extraction of the features is given below.

#### 3.2.1. Baseline of FHR

FHR baseline (BL) was estimated iteratively based on the assessment of an initial virtual baseline within a 10 min window [26]. The effect of this initial calculation of baseline is nullified due to the iterative calculation of the adjusted baseline, which ultimately approaches the original baseline of the FHR signal. Each stage of iteration removes accelerations and decelerations. This algorithm is applied in a 10 min window with 3 minutes of overlap. If within each window is an identifiable baseline of duration more than 2 min (not necessarily contiguous), then the baseline value is accepted; otherwise, it is rejected. Baseline was classified as Normal, Bradycardia, and Tachycardia based on the amplitude of FHR.

#### 3.2.2. FHR Variability

A given FHR signal was disintegrated to its component frequencies, where each frequency is represented by its amplitude expressed in bpm. FHR variability (FHRV) was calculated in each discreet 10 min slice by computing the deviation of the FHR amplitude from the baseline within a one minute segment. If the number of cycles in each segment is more than one, then that segment has an identifiable baseline variability value. FHR variability (FHRV) is the standard deviation of the FHR values at each point. Summation of the value of variability for all the segments where the number of cycles exceeds one, gives the variability of the FHR for a 10 min window, and the average FHRV of all the 10 min windows is the variability of the CTG trace [27].

#### 3.2.3. Acceleration

Three points are used to identify each acceleration (Ac): the beginning, where the fetal heart rate crosses the baseline; the peak of Ac; and the end, i.e., where FHR again crosses the baseline. The duration of each deviation and the peak are noted. If the duration is between 15 sec and 10 min and the difference between the peak and the baseline is greater than 15 bpm, then the deviation was identified as acceleration. Acceleration that lasts for more than 10 min is considered a baseline change. Acceleration was classified as either present or absent [28].

#### 3.2.4. Deceleration

Besides the FHR baseline, the next important feature is the type of deceleration, which is a major indicator of fetal health status. The classification of deceleration plays a major role in the classification of FHR patterns into the three-tier system, i.e., normal, suspicious, and pathological. The spatial and temporal relationship of FHR with the uterine contraction pressure (UCP) of the mother can help classify the deceleration as Early, Late, or Variable. The authors proposed a fuzzy-logic-based method to identify a deceleration, extract the event points of both FHR and UCP, and finally classify the decelerations.

The algorithm proposed by the authors to estimate the deceleration assesses the width as well as the amplitude of any negative deviation from the baseline and identifies it as deceleration if both the amplitude and the width conforms to the definition provided by the different international obstetric bodies. Every deceleration, De, was identified using three points—the start, where the fetal heart rate falls below the baseline; the nadir of De; and the end, i.e., where FHR again goes above the baseline. The duration of each De was noted.

According to NICHD guidelines, the length of deceleration should be at least 15 s but not more than 10 min, in which case it is considered a baseline change. The difference between the nadir and the baseline should be at least 15 bpm. However, in clinical scenarios may not conform to such a strict definition. Therefore, the authors used a fuzzy-logic-based approach to identify the length and width in order to define a period with negative deviation from the baseline as deceleration. Finally, the classification of deceleration was performed using machine-learning-based techniques.

### 3.3. Classification of FHR for First Stage of Labor

The dataset is $D=\left\{d_{1},d_{2},\ldots .,d_{n}\right\}$, where each data sample $d_{i}$ has a feature set consisting of 11 features $X=\left\{x_{1},x_{2},\ldots .,x_{10}\right\}$. The features are listed in Table 1.

A total of 399 cases from the CTU-UHB dataset were used for the experiment. Six clinicians separately annotated the data. The final annotation was performed by taking a majority vote. The CTG trace is classified as Normal (1), Suspicious (2), and Pathological (3). The annotated data was used as input to the classifiers. The input vector is represented as:

$$I\;=\;\left[\begin{array}{cccc} v_{11} & . & . & v_{1n}C_{k}\\ . & . & . & .C_{k}\\ . & . & . & ..\\ v_{m1} & . & . & v_{mn}C_{k} \end{array}\right]$$

where the feature value for each data sample is represented as $v_{ij}$ and $C_{k}$, k=1,2,3 denotes the class of each sample. The four classifiers used for building different classification models are discussed below.

#### 3.3.1. Random Forest (RF)

RF is known to provide a higher level of accuracy. It uses the bagging method, which is a combination of learning models that provides a more accurate and stable outcome. It searches for the best feature among a random subset of features, leading to a wide variety that generally results in a better model. Hence, it is a suitable model for predictive modelling. The associated algorithm is given in Algorithm 1:
**Algorithm 1:** FHR Classsification using Random Forest*Input: *$S_{i}\subseteq \left\{\left(d_{1},y_{1}\right),\ldots .,\left(d_{n},y_{n}\right)\right\}$*Step1: Perform row and column sampling**Step2: Decision tree *$DT_{i}$* for each *$S_{i}$*Step3: Prediction*$P_{i}$ ←* output of each*$S_{i}$*Output: *P←* majority vote of *$P_{1},\ldots .,P_{i}$

#### 3.3.2. Multilayer Perceptron (MLP)

MLP offers approximate solutions for complex problems. The input layer has 10 features along with weights, $w_{i}$, and the annotated class of the sample as input units. The output layer has one output unit. There are three hidden layers with sigmoid as the activation function. The use of the sigmoid function for the hidden layers is appropriate for the problem since we need to consider a soft decision boundary. The associated algorithm is given in Algorithm 2:
**Algorithm 2:** FHR Classsification using Multi Layer Perceptron*Input: *$X=\left[x_{1},\ldots .,x_{10}\right]$*, class labels, initial weight vector *$w={\left[w_{i}\right]}^{T}$*Step 1: Weighted sum of the input features*$g\left(x\right)\leftarrow \overset{n10}{\underset{i=1}{\sum }}w_{i}x_{i}$*Step2: Pass the value to the sigmoid activation function f.**Step3: Produce the output*y←f(g(x))*Step4: Compute the error *$e_{i}=h_{i}-y_{i}$*Step5: Adjust*w*to minimize *$e_{i}$.*Output:*$\hat{y}=\frac{\sum_{i=1}^{10}w_{i}f_{i}}{\sum_{i=1}^{10}w_{i}}$

#### 3.3.3. Support Vector Machine

Training samples are mapped to high-dimensional feature space. SVM can be easily extended into complex classification problems with more than two classes. In the current classification problem it is not always possible to find the hard boundary between the classes. SVM is capable of dealing with soft boundaries for multiclass classification problems by introducing a slack variable that allows the input $d_{i}$ to be closer to the hyperplane. The associated algorithm is given in Algorithm 3:
**Algorithm 3:** FHR Classsification using Support Vector Machine*Input: *$\Delta =\left\{\left(d_{1},y_{1}\right),\ldots .,\left(d_{n},y_{n}\right)\right\}$*, class labels*$C=\left[c_{1}|,c_{2},c_{3}\right]$*, initial weight vector *$w={\left[w_{i}\right]}^{T}$*, hyper-parameters, training set *T*Step1: Define the hyperplane *$w^{T}*d+b\leftarrow 0$*Step3: Maximize the margin*$w*d+b\vee \frac{w}{\left|w\right|\vee \leftarrow \frac{1}{\left|w\right|\vee }}$*between the hyperplane and the plane median**Step4: Minimize *|w|∨* to maximize the margin**Output: *$w_{T+1}$

#### 3.3.4. Bagging

Bagging is a commonly used ensemble method that creates several training sets using boot-strapping. It is useful in medical data analysis because it constructs a classification tree by bootstrapping the training data and then aggregating the predictions to produce the outcome. During bootstrapping, multiple training sets are created choosing the samples randomly and repeatedly. The training of each learner results in the generation of multiple learning models. This improves the accuracy of the prediction. The associated algorithm is given in Algorithm 4:
**Algorithm 4:** FHR Classsification using Bagging*Input: *$\Delta =\left\{\left(d_{1},y_{1}\right),\ldots .,\left(d_{n},y_{n}\right)\right\}$*, class labels, base learning algorithm *L*, number of learning rounds *j*, training set *T*Step1: *$\Delta_{t}\leftarrow$* Bootstrap *Δ*Step3: *$h_{t}\leftarrow L\Delta_{t}$*Step4: *j←j+1*Step5: Repeat steps 1–3 till j covers the entire training set *T*Output:*$\hat{y}\left(d\right)=argmax\overset{T}{\underset{j=1}{\sum }}\left(y=h_{t}\left(d\right)\right)$

For the purpose of classification, the dataset was split into k-number of folds where each fold is used as a testing set at some point, whereas other folds are used for training. The value of k was set to 5. Rather than splitting the data into train–test sets, the cross validation was chosen because it gives a less biased estimate of the model skill. The algorithm for the classification with k-fold cross validation is given in Algorithm 5:
**Algorithm 5:** Algorithm for the k-fold cross validation*Input: *$D=\left\{d_{1},d_{2},\ldots .,d_{n}\right\}$*, class labels**Step1: Initialize *i←1,k←5*Step2: Split *D* into k-folds**Step3: In iteration*i*, the *$i^{th}$* fold is used for testing and the rest are used for training**Step4: *i←i+1*Step5: Repeat steps 3–4 while *i≤k*Step6: Evaluate model on test score*

### 3.4. Data Augmentation

A large dataset is important for the improved performance of a machine learning model. However, the existing dataset is imbalanced. Thus, to increase the diversity of the data, we have performed data augmentation using the synthetic minority oversampling technique (SMOTE). The normal and suspicious CTGs are the majority class $C^{-}$, whereas pathological CTGs are the minority class $C^{+}$. The total synthetic samples are:

*S* = (*C* − *C*^+^) × *C* + *C^−^*

The size of the dataset after augmentation was 1968. The same set of classifiers were used with the augmented data and the accuracy was noted for both the training and testing data. SMOTE is applied only to the training dataset, whereas the test dataset remains unchanged to correctly represent the original data.

### 3.5. Classification of the Second Stage of Labor

Another complication associated with the classification of CTG is the existence of two different stages of labor. In the first stage, cervical dilation occurs with regular contractions. The second stage is characterized by active pushing, during which uterine contraction is more frequent and so are the decelerations. Interpretation of the parameter values also change during this stage. While building a robust classification system most researchers consider either one of the labor stages or make no distinction between the stages. The first approach is methodologically correct but does not take into consideration the change in feature values that might lead to a better recognition of the fetal state. The second approach can lead to erroneous classification because it does not consider the radical changes in FHR parameter dynamics. In this work, the authors applied the classification and feature extraction algorithms to the second stage as well, while taking into consideration the changed dynamics in this stage.

## 4. Results and Discussion

The analysis of the result can be categorized into four categories:

- Adequacy of the dataset.
- Outcome of the classification and the comparison of the performance of the classifiers.
- Agreement between the manual annotation and the classifier-based outcome.
- Comparison of the classifiers’ performance for different stages of labor.

### 4.1. Kaiser–Meyer–Olkin (KMO) and Bartlett’s Test

**Figure 2:**
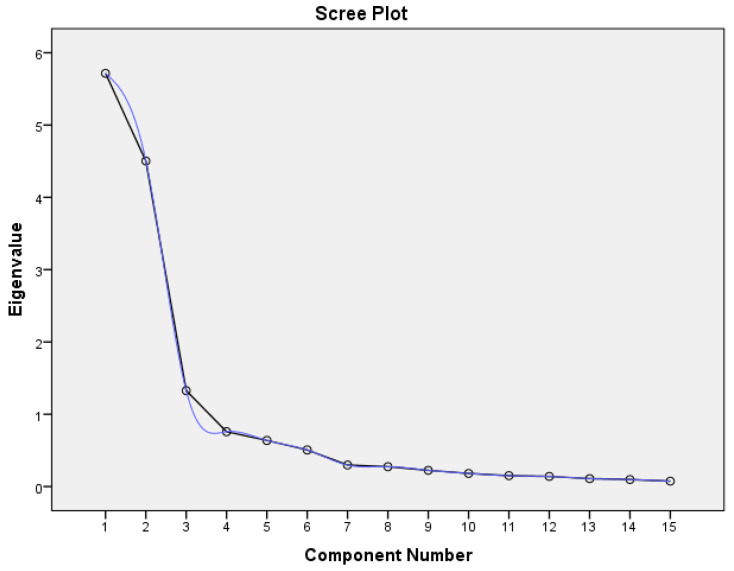
The scree plot begins to flatten from the 9th feature.

Sample size is the most important factor to be considered to determine if the data is appropriate for the experiment. Sample size should be more than 100 [28] and at least 300 for factor analysis [29]. Also, the number of samples should be a least five times more than the number of features [30]. KMO was conducted to measure the sample adequacy, which is used to compare the magnitudes of the observed correlation coefficients in relation to the partial correlation coefficients. KMO ≈ 1 is considered ideal; KMO < 0.5 is unacceptable. Bartlett’s test of sphericity is used to establish that there is no redundancy between the features, i.e., an ideal value for Bartlett’s sphericity means that the null hypothesis of uncorrelated features is rejected. Both the values are shown in Table 2. A scree plot is plotted to find out the number of features that can be retained by recognizing the point of inflexion. The number of features before the inflexion represents the important features to be extracted for the factor analysis. The plot is shown in Figure 2.

According to the scree plot, all the features up to the ninth feature are important for the classification. For the classification, the first nine features are adequate. The 10th and 11th features have been added to ensure the robustness of the system.

### 4.2. Sensitivity Analysis of the 5-Fold Cross-Validation

**Figure 3:**
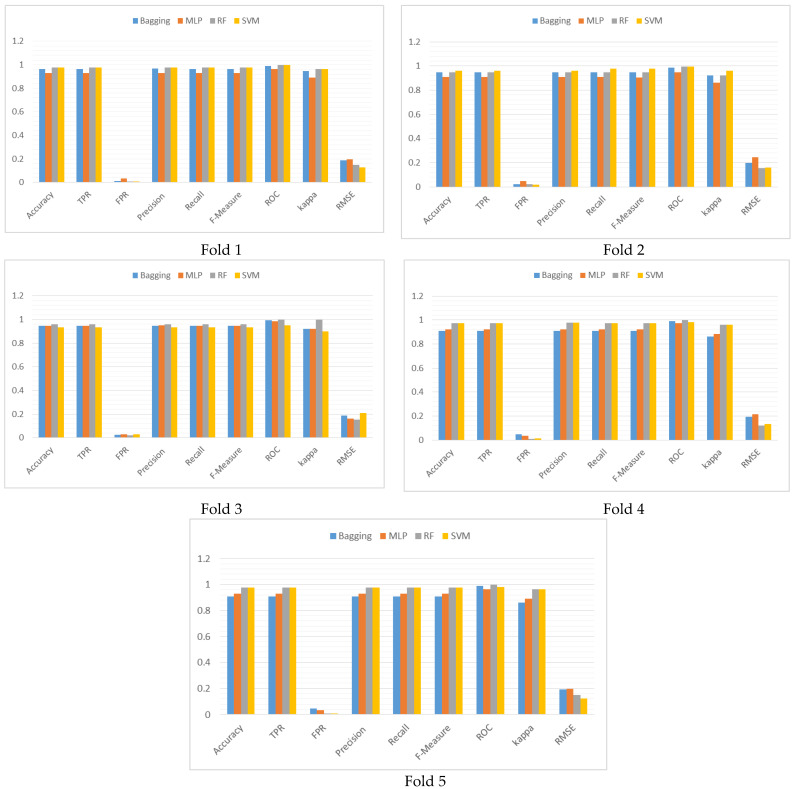
Graphical representation of the performance comparison of the classifiers for each fold of the 5-fold cross validation in terms of the performance metrics.

**Figure 4:**
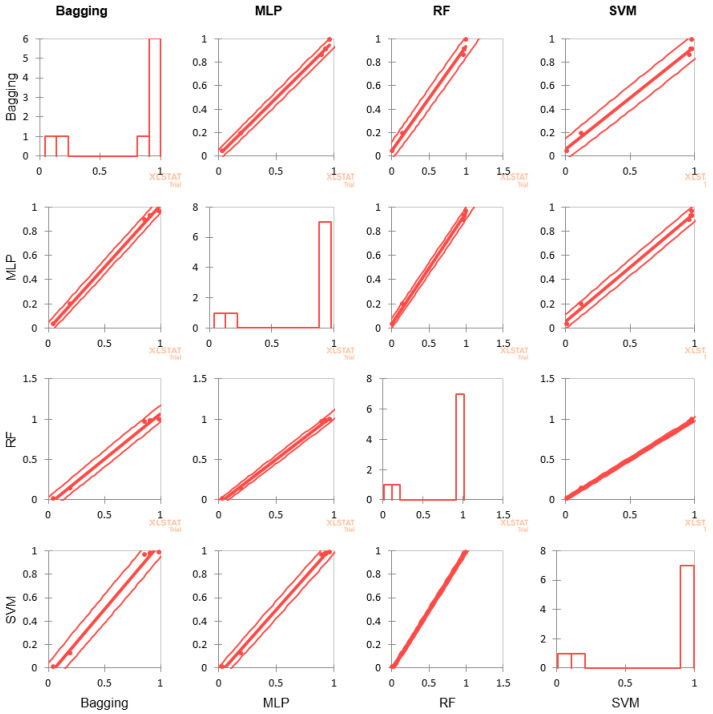
Scatter plot of the correlation analysis for the four classifiersestablishes how well the chosen value of k matches across the different algorithms.

The authors have evaluated the choice of k value for the k-fold cross validation. Distribution of the outcome for each fold for each different classifiers are compared to check the robustness of the chosen configuration of k. Values of the performance metrics set:

(2) $$\begin{array}{cc} M & =\{Accuracy,TPR,FPR,Precision,Recall,F\\ & -Measure,ROC,kappa,RMSE\} \end{array}$$

were compared and shown graphically in Figure 3. The correlation matrix is shown in Table 3, and graphical representation of the correlation is given in Figure 4.

Metric measurements of the classifiers for each fold are almost equal except for MLP. Correlation of the performance outcome in Table 3 suggests high correlation among the classifier performance in fold 1, whereas in other folds, except for the highlighted, correlations are very high. The scatter plot of Figure 4 has very few outliers and performance measures for SVM and RF are almost identical. Thus, the choice of k = 5 is appropriate for the purpose.

The correlation test not only established the appropriateness of the chosen test harness, but also established that the value of k performed equally well across the different classifiers. The scatter plot has revealed how closely the performance of the classifiers match when compared pairwise.

### 4.3. Model Performance Measure

Metrics of the model performance measures for the classifiers are given in Table 4. Though accuracy is normally considered a measure for evaluating the performance of a classifier, it can be ambiguous if the dataset is not balanced. In such cases, more emphasis is on the majority class, which makes it difficult for the classifier to perform well on the minority class. The parameter values for all the classes are comparable, though the performance of the RF and SVM are better.

#### 4.3.1. Combined Performance Measure

The authors also establish that the proposed model balances between false positive rate (FPR) and false negative rate (FNR). Such measures cannot be provided by model performance metrics. Hence, the values of combined performance measures are given in Table 5.

Since G-mean > 0.9 for all the classifiers, the performance of the classifiers in identifying positive classes is very high. However, the values of DP < 0.2 indicate that the classifiers have limited capacity in distinguishing between normal and abnormal (suspicious and pathological) classes. The average accuracy or the balanced accuracy for both the major and minor classes are fair. MCC values indicate that the correlation between classifier prediction and visual prediction are high for SVM and bagging and moderate for MLP and RF. Cohen’s kappa > 0.8 is in almost perfect concordance with the classifier model prediction and the classified class. All the classification models are good at avoiding misclassification because Youden’s index > 0.8.

#### 4.3.2. Model Performance Comparison

**Figure 5:**
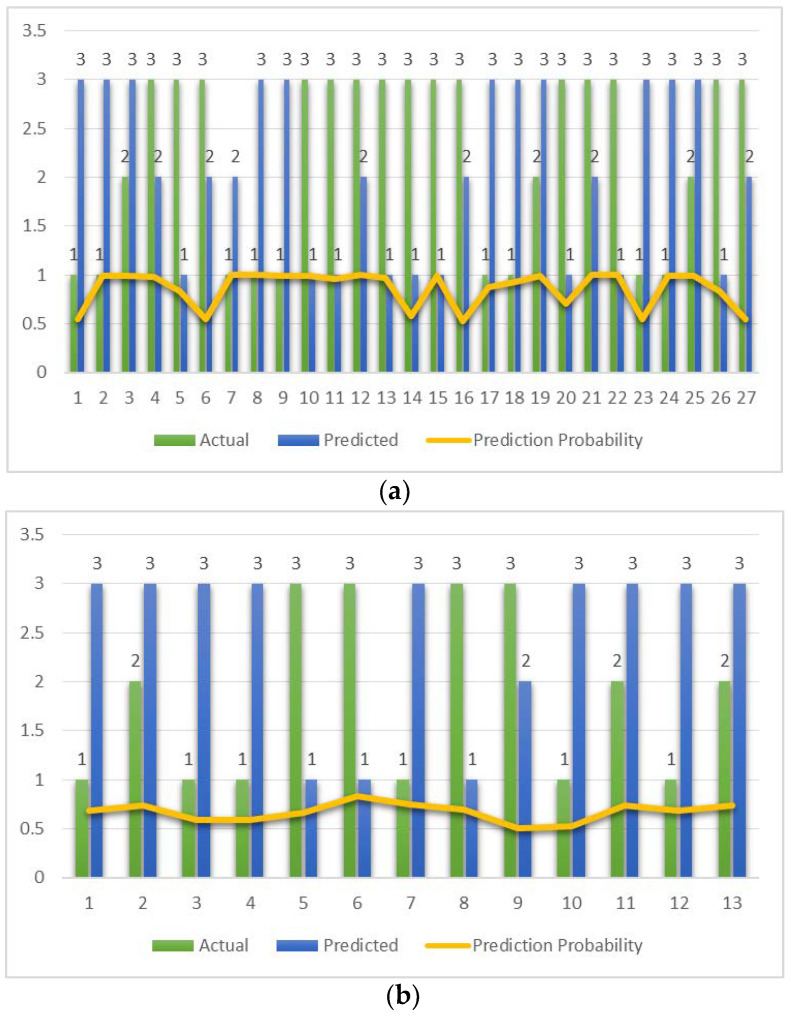

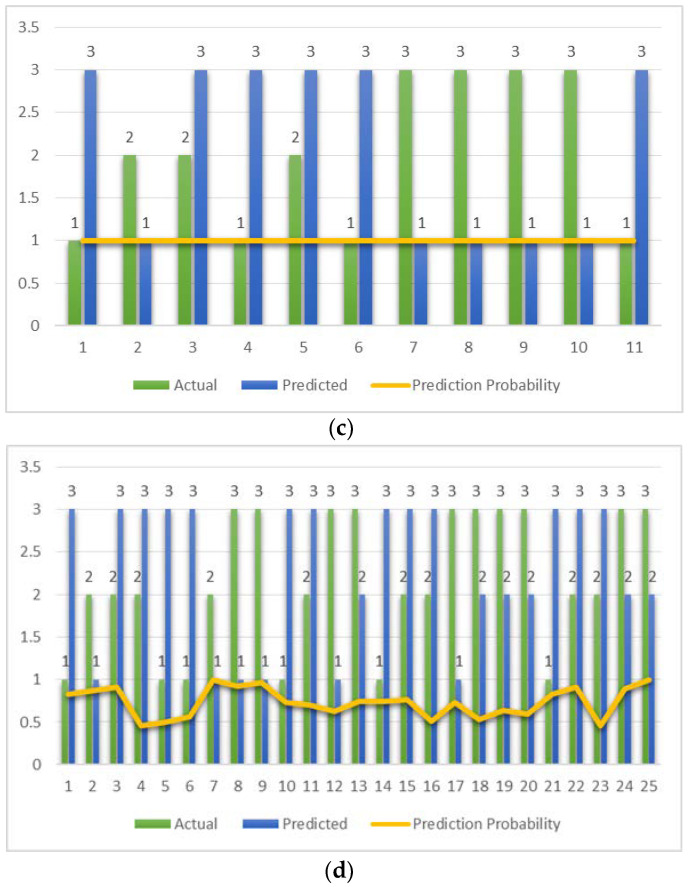
Data points where actual and predicted classifications differ for each of the classifiers. The prediction probability is shown as a yellow line. (**a**) MLP. (**b**) RF. (**c**) SVM. (**d**) Bagging.

The number of data points where actual and predicted classifications differ along with the actual and predicted class are shown in Figure 5, along with the prediction probability. The prediction probability summarizes how well the predicted class matches the actual class.

The maximum number of misclassifications is observed with MLP and bagging with 27 and 25 data points, respectively. Prediction probability is unvarying for SVM.

#### 4.3.3. AUC-ROC Curve

**Figure 6:**
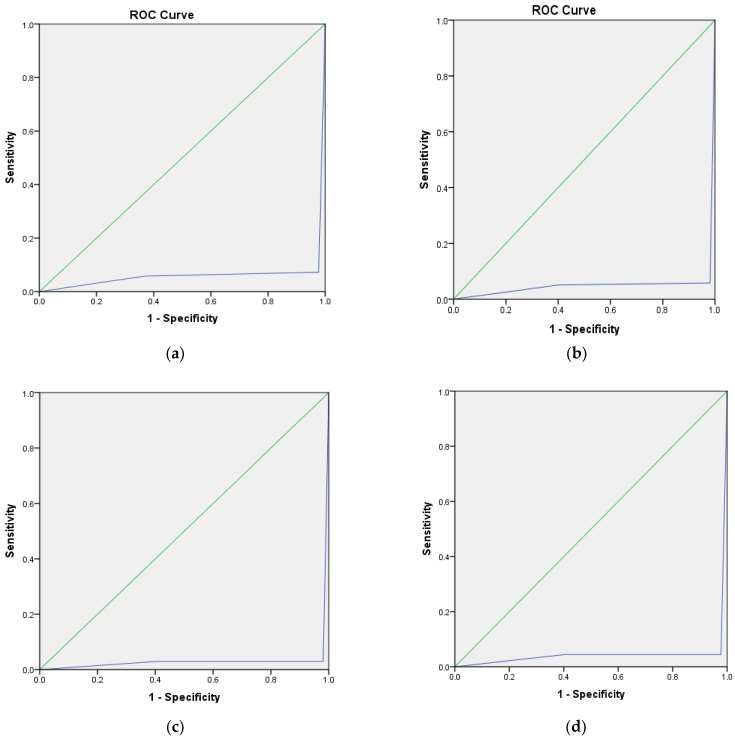
ROC curve for the four classifiers. Predicted has at least one tie between the positive actual state group and the negative actual state group. (**a**) MLP, AUC-ROC: 0.966. (**b**) RF, AUC-ROC: 0.997. (**c**) SVM, AUC-ROC: 0.966. (**d**) Bagging, AUC-ROC: 0.989.

Since it is a multi-class classification problem, the AUC-ROC was used to visualize the performance of the classifiers. The ROC curve is plotted with TPR (sensitivity) against the FPR (1—specificity) in Figure 6. The AUC values are 0.966, 0.997, 0.966, and 0.989 for MLP, RF, SVM, and bagging, respectively. The AUC-ROC value greater than 0.9 indicates high discrimination capacity for all the classifiers for all the classes. All the classifiers exhibit very high discrimination capacity.

### 4.4. Performance Comparison of the Classifiers with Clinicians’ Annotation

Performance of the four classifiers are compared with the visual estimate using covariance correlation and a contingency table. A total of 399 observations were annotated as Normal (1), Suspicious (2), and Pathological (3).

#### 4.4.1. Covariance Correlation

**Figure 7:**
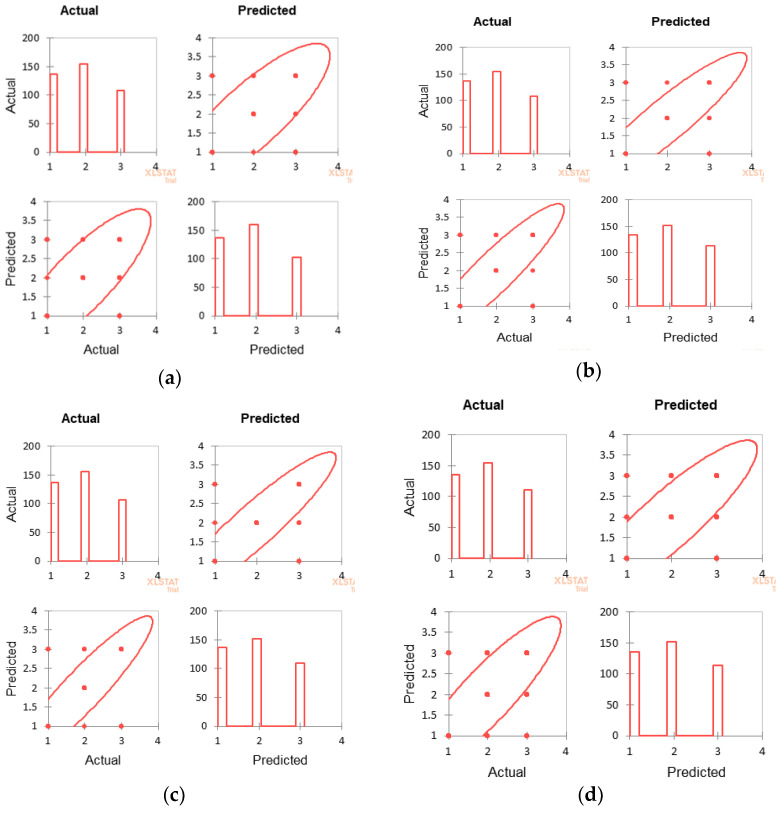
Scatter plots show the difference between the actual and predicted classifications. The bar charts show the number of instances in each class and the scatter plots show the number of outliers in each class for all four classifiers. (**a**) MLP. (**b**) RF. (**c**) SVM. (**d**) Bagging.

The degree of association between the actual and predicted values are analyzed and the results for all four classifiers are given in Table 6; the scatter plots are shown in Figure 7. All the models show almost similar standard deviation between the actual and the predicted values.

The scatter plot shows that many traces that had been annotated as Suspicious (2) or Pathological (3) were predicted as Normal (1) by all the classifiers. On the other hand, actual Pathological ones have been predicted by the classifiers as Normal or Suspicious. This anomaly in the prediction can cause serious damage to the fetus. The degree of disagreement is most evident with the bagging classifier. Also, this contradicts the result of Table 5 where Youden’s Index indicates the avoidance of misclassification by all four classifiers. This leads us to hypothesis test.

#### 4.4.2. Contingency Table

Since we are dealing with categorical data, the contingency table is used for hypothesis testing along with estimation of model parameters. Contingency dals with:

- Hypothesis testing that verifies whether there is any association among the actual and predicted classifications or whether they are independent.
- Which model provides the better option for the overall classification of CTG?

The null hypothesis H0: The rows and the columns of the table are independent. The alternate hypothesis Ha: There is a link between the rows and the columns of the table.

The observed frequencies for the actual and predicted classifications are shown in Table 7. The result of the test for independence or the chi-squared test for degree of freedom (DF) = 4 and α = 0.05 are shown in Table 8. As the computed p-value is lower than the significance level alpha = 0.05, one should reject the null hypothesis H0 and accept the alternative hypothesis Ha, i.e., the annotated class and the predicted class are related; however, this test does not reveal the difference between the two methods of classification and the Bland–Altman test was performed.

#### 4.4.3. Bland–Altman Analysis

**Figure 8:**
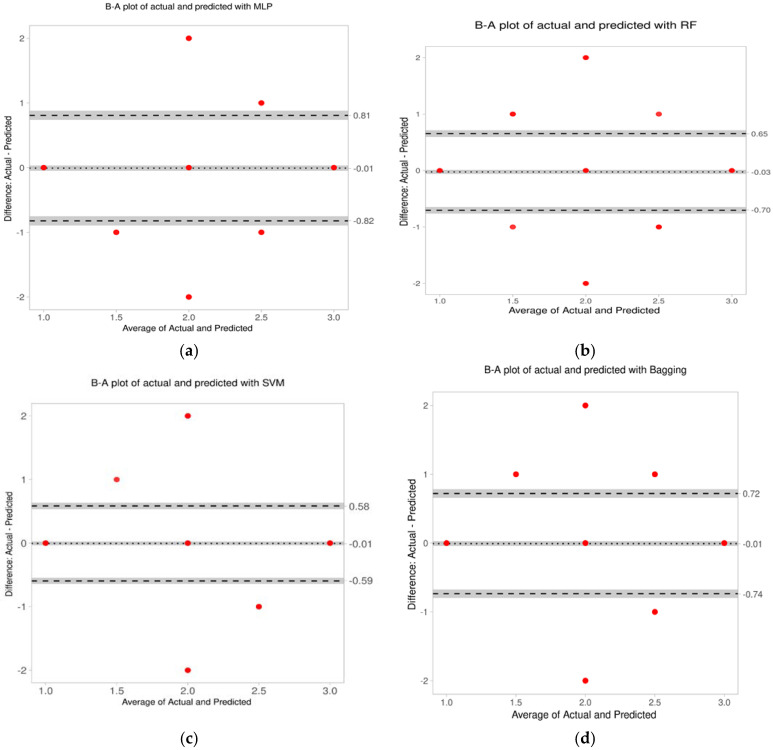
Bland–Altman plot of the actual and predicted classifications for all the classifiers. (**a**) For MLP, the average difference is −0.01 and the limits of agreement: −0.82 and 0.81. (**b**) For RF the average difference is −0.03 and the limits of agreement: −0.7 and 0.65. (**c**) For SVM, the average difference is −0.01 and the limits of agreement: −0.59 and 0.58. (**d**) For bagging the average difference is −0.01 and the limits of agreement: −0.74 and 0.72.

Bland–Altman (B-A) analysis was performed to find the difference between the actual and predicted classifications [31] The difference between the two methods was measured by constructing limits of agreement (LoA). A graphical approach is used to verify the assumption of normality of differences. The lack of agreement is summarized by computing the bias, estimated by the mean difference and the standard deviation of the differences. Parameters of the B-A analysis are given in Table 9, and the plots for it shown in Figure 8. The number of outliers is insignificant, and the limits of agreement are also within acceptable limits.

The bias is insignificant because the line of equality is within the CI of the mean difference. Thus, no method over- or under-estimates compared with the actual annotation. Though the limits of agreement (LoA) for the difference is within an a priori limit, the LoA is narrowest at 0.5 and 0.6 for SVM and RF, respectively. The agreement of the upper and lower limits for SVM and RF are also smaller compared with the other classification methods. Thus, the correlation between the actual annotation and the predictions by SVM and RF are both high.

### 4.5. Result with Augmented Data

**Figure 9:**
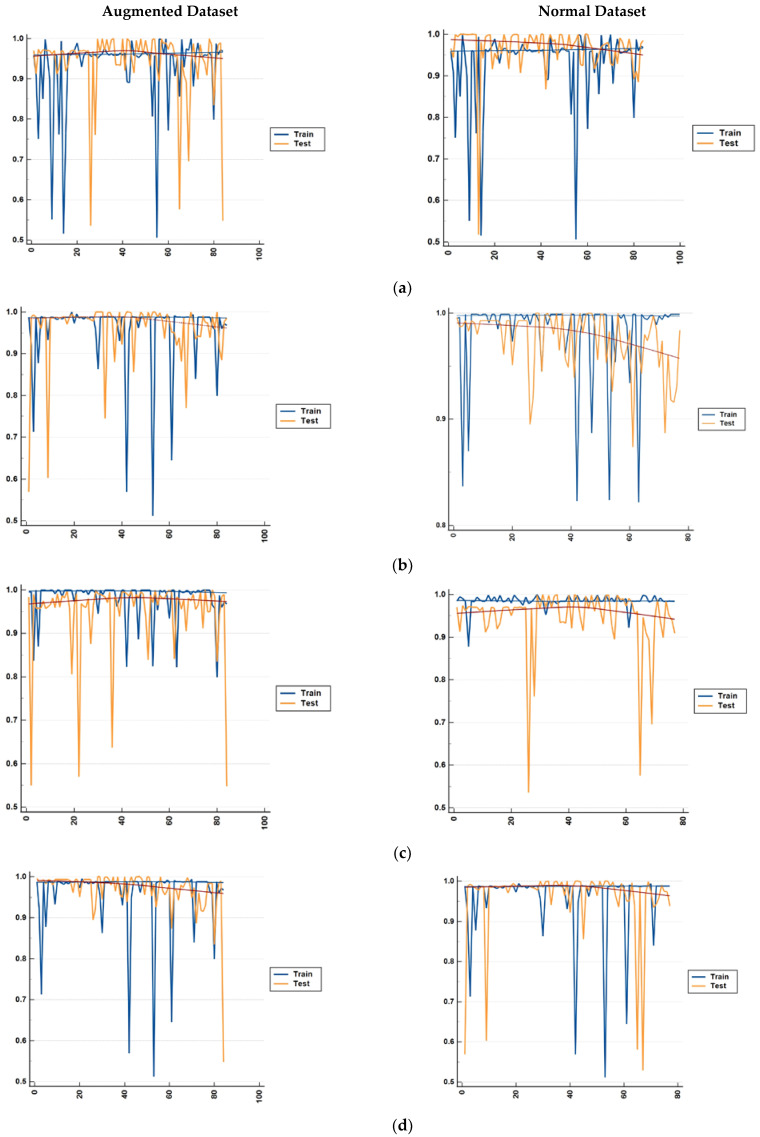
Comparison of the differences in accuracy for both the augmented and normal datasets for both the training data and test data. The result is shown for each fold of the 5-fold cross validation for (**a**) bagging, (**b**) random forest, (**c**) MLP, and (**d**) SVM.

Training models on limited data leads to overfitting, which is exhibited by high accuracy on the training dataset but a lack of generalization to the test data. This difference for both sets of data is shown in Figure 9. For both sets of data, there are differences in accuracy for the training and test data. This could mean overfitting, however, since the difference between the accuracy at each fold is below 4% the overfitting can be ignored. No improvement in accuracy is observed with the augmented data.

### 4.6. Class-Wise Accuracy Estimation for Stage 1 and Stage 2 Labor

Feature values from the second stage of labor were fed into the same set of classifiers and the overall accuracies were 86.2%, 92.6%, 92.3%, and 88.2%, respectively, for MLP, RF, SVM, and bagging. The accuracy of each class for the two stages of labor are given in Table 10.

### 4.7. Comparison with Other Works

Some of the recent works along with their outcomes are listed in Table 11.

## 5. Conclusions

The primary objective of this work was to develop a robust classification model for CTG that can identify fetal distress at both stage 1 and stage 2 of labor. The accuracy for both stages for each class is presented in Table 10. The difference in accuracy was mainly due to the presence of noise and motion artefacts in the FHR signal in stage 2 of labor. A sample of adequate size had been used for the experiment as established by the KMO and Bartlett’s Test. The experiment was repeated with augmented data, but the deviation in accuracy for each fold was less than 4%. The sensitivity and the specificity for different classifiers are in the ranges 92.7–96.4% and 92.8–98.4%, respectively. The AUC, which is greater than 0.9 for all classifiers, indicated their high discrimination capacity. The combined performance measure reveals that the discriminant power of SVM and RF were highest at 1.744 and the maximum number of misclassifications were with MLP and bagging.

The authors used annotation by six clinicians, and the final annotation for each record was chosen using majority voting. The Bland–Altman plot shows the strong agreement between the clinicians’ annotation and the classification obtained with SVM and RF. Performance measures for the four classifiers and the performance in comparison with clinicians’ annotations are summarized in Table 12. SVM and RF had improved classification performance. The performance of the classifiers varies in different stages of labor. In the second stage of the labor, the accuracy of the classifiers is comparatively low; however, SVM and RF still performed better than the other two. The accuracy, sensitivity, specificity, and AUC, etc., are sufficiently high for both SVM and RF. These two classifiers also exhibited a high level of agreement between the system and the human annotators.

The other feature-based approaches to the automation of CTG classification that were based on weak annotation did not achieve high AUC, sensitivity, and specificity. In fact, with weak annotations, the systems showed little improvement over the visual interpretation.

Features used in stage 1 of labor are typically embedded in clinical exercise. However, features such as deceleration and average baseline are no longer applicable for stage 2. Since there is active maternal pushing, the decelerations are recurrent and large. The data is burstier and hence there is a dwindling of the accuracy level. In subsequent work, the authors intend to implement the classification of CTG in the second stage of labor after removing the noise and artefacts.

## Figures and Tables

**Table 1:** Description of the feature set for the classification of CTG.

Feature Number	Feature Set	Description
1	Baseline (BL)	Baseline of the FHR
2	Baseline type (B_type)	Classification of BL as Normal, Bradycardia, or Tachycardia
3	Variability (V)	Variability of FHR baseline
4	Variability type (V_type)	Classification variability as Absent, Minimal, Moderate, or Marked
5	Acceleration (Ac)	Acceleration of FHR
6	Number of accelerations	Total number of accelerations present in a CTG trace.
7	Number of Early decelerations	Number of Early decelerations present in a CTG trace
8	Number of Late decelerations	Number of Late decelerations present in a CTG trace
9	Number of Variable decelerations	Number of Variable decelerations present in a CTG trace
10	Sinusoidal heart rate (SHR)	Sinusoidal heart rate pattern present or not. SHR in a CTG trace is denoted as Present, Absent, or Undetermined.
11	Stage of labor	Stage of labor can be normal, stage 1, or stage 2.

**Table 2:** KMO and Bartlett’s test. A KMO value greater than 0.8 indicates the presence of a strong partial correlation among the features and the number for the significance of Bartlett’s test is less than 0.05.

KMO and Bartlett’s Test		
Kaiser-Meyer-Olkin Measure of Sampling Adequacy.		0.867
Bartlett’s Test of Sphericity	Approx. Chi-Square	5612.158
	df	105
	Sig.	0.000

**Table 3:** Correlation matrix of the classifiers depicting the degree of correlation in terms of M.

**Fold 1**				
**Variables**	**Bagging**	**MLP**	**RF**	**SVM**
Bagging	**1**	0.888	0.953	0.953
MLP	0.888	**1**	0.931	0.931
RF	0.953	0.931	**1**	1.000
SVM	0.953	0.931	1.000	**1**
**Fold 2**				
Bagging	**1**	0.888	1.000	0.832
MLP	0.888	**1**	0.888	0.615
RF	1.000	0.888	**1**	0.832
SVM	0.832	0.615	0.832	**1**
**Fold 3**				
Bagging	**1**	0.888	0.538	1.000
MLP	0.888	**1**	0.478	0.888
RF	0.538	0.478	**1**	0.538
SVM	1.000	0.888	0.538	**1**
**Fold 4**				
Bagging	**1**	0.901	0.742	0.742
MLP	0.901	**1**	0.931	0.931
RF	0.742	0.931	**1**	1.000
SVM	0.742	0.931	1.000	**1**
**Fold 5**				
Bagging	**1**	0.901	0.742	0.742
MLP	0.901	**1**	0.931	0.931
RF	0.742	0.931	**1**	1.000
SVM	0.742	0.931	1.000	**1**

**Table 4:** Metric values of the model performance measure.

Classifier	Accuracy	Sensitivity	Specificity	Precision
MLP	0.927	0.927	0.962	0.928
RF	0.967	0.964	0.984	0.968
SVM	0.964	0.964	0.983	0.966
Bagging	0.936	0.936	0.968	0.936

**Table 5:** Metrics values of the combined performance measure.

Classifier	G-Mean	Discriminant Power (DP)	Balanced Accuracy	Matthew’s Correlation Coefficient (MCC)	Cohen’s Kappa	Youden’s Index
MLP	0.944	1.382	0.446	0.784	0.889	0.889
RF	0.974	1.774	0.474	0.696	0.950	0.948
SVM	0.973	1.774	0.474	0.970	0.946	0.947
Bagging	0.952	1.459	0.453	0.904	0.902	0.904

**Table 6:** Correlation between the actual and the predicted values for the classifiers.

Classifier	Variable	Mean	Std. Deviation
**MLP**	Actual	1.927	0.781
	Predicted	1.912	0.770
**RF**	Actual	1.927	0.781
	Predicted	1.947	0.786
**SVM**	Actual	1.927	0.778
	Predicted	1.932	0.785
**Bagging**	Actual	1.937	0.782
	Predicted	1.945	0.787

**Table 7:** Observed frequencies for actual and predicted classification of CTG.

Classifier	Actual/Predicted	1	2	3
**MLP**	1	128	1	8
	2	0	151	3
	3	9	8	91
**RF**	1	131	0	6
	2	0	151	3
	3	3	1	104
**SVM**	1	132	0	4
	2	1	152	3
	3	4	0	103
**Bagging**	1	129	0	6
	2	2	145	7
	3	4	6	100

**Table 8:** Contingency table showing the chi-squared values and the p-value for each classifier.

Classifier	Chi-Squared (Observed Value)	Chi-Squared (Critical Value)	*p*-Value
**MLP**	624.194	9.488	<0.0001
**RF**	717.664	9.488	<0.0001
**SVM**	723.633	9.488	<0.0001
**Bagging**	650.510	9.488	<0.0001

**Table 9:** Parameters of the Bland–Altman analysis.

**MLP**			
**Parameter**	**Value**	**95%CI lower limit**	**95%CI upper limit**
Difference	0.02	−0.03	0.06
Upper Limit of Agreement	0.89	0.82	0.97
Lower Limit of Agreement	−0.86	−0.94	−0.79
Intercept	−0.02	−0.14	0.11
Slope	0.02	−0.04	0.08
**RF**			
**Parameter**	**Value**	**95%CI lower limit**	**95%CI upper limit**
Difference	−0.02	−0.05	0.01
Upper Limit of Agreement	0.60	0.55	0.65
Lower Limit of Agreement	−0.64	−0.69	−0.59
Intercept	−0.01	−0.09	0.08
Slope	−0.01	−0.05	0.03
**SVM**			
**Parameter**	**Value**	**95%CI lower limit**	**95%CI upper limit**
Difference	−0.01	−0.03	0.02
Upper Limit of Agreement	0.58	0.53	0.64
Lower Limit of Agreement	−0.59	−0.65	−0.54
Intercept	0.01	−0.07	0.09
Slope	−0.01	−0.05	0.03
**Bagging**			
**Parameter**	**Value**	**95%CI lower limit**	**95%CI upper limit**
Difference	−0.01	−0.04	0.03
Upper Limit of Agreement	0.72	0.66	0.78
Lower Limit of Agreement	−0.74	−0.80	−0.67
Intercept	0.01	−0.09	0.11
Slope	−0.01	−0.06	0.04

**Table 10:** Accuracies for each class for each of the four classifiers.

Classifier	Stage of Labor					
	Stage 1			Stage 2		
	Normal	Suspicious	Pathological	Normal	Suspicious	Pathological
MLP	93.4%	98%	84.3%	89.3%	88.7%	79.2%
RF	95.6%	98%	96.3%	92.8%	89.3%	92.3%
SVM	97%	97.4%	96.3%	91.2%	90.6%	92.7%
Bagging	95.6%	94.2%	90.9%	89.8%	86.5%	86.7%

**Table 11:** A comparison with some of the recent works for the classification of CTG.

Author	Dataset	Number of Samples	Annotations	Method Used	Outcome
Comert et al. (2017) [12]	UCI Machine Learning Repository	2126 with 21 features and 3 classifications.	3 obstetricians	ANN with 21 inputs, 10 hidden layer, 3 output nodes. Tan sigmoid and softmax functions were used in the hidden layer.	Sensitivity = 97.9% Specificity = 99.7%
Signorini et al. (2019) [32]	Ob-Gyn Clinics at the Azienda Ospedaliera Universitaria Federico II, Napoli, Italy	60 normal and 60 IUGR samples	Normal and IUGR	Linear Logistic Regression, SVM, Naïve Bayes, Random Forest, RIDGE Regression	Sensitivity = 83.8–86.7% Specificity = 76.7–87.1%
Rahmayanti et al.(2021) [24]	UCI Machine Learning Repository	2126 with 21 features and 3 classifications	3 obstetricians	ANN and LSTM with ReLU activation and Adam optimizer.	Accuracy = 9–37%
Ogasawara et al (2021). [23] )	CTG data from Keio University hospital	380 with 2 classifications—normal and abnormal	Umbilical artery pH value and apgar score	Deep Neural Network (DNN) with 3 convolution layers. Normal and abnormal are 2 outputs.	AUC-ROC = 0.73 ± 0.04
Petroziello et al (2019) [22].	Oxford archive	35,429 CTG data	Umbilical artery pH value and apgar score	Multimodal-CNN	Only 1% improvement in FPR and 19% improvement in TPR compared to normal clinical practice.
Zhao et al. (2019) [33]	CTU-UHB dataset for CTG	552 CTG data	9 obstetricians	Deep CNN with 8 layers	Sensitivity = 93.45%, Specificity = 91.22%
**Current Work**	**CTU-UHB dataset for CTG**	**399 data with three classifications**	**6 obstetricians**	**Best result with Random Forest**	**Sensitivity = 96.4%** **Specificity = 98.4%**

**Table 12:** Performance measure of the classifiers in terms of various statistical evaluation metrics and classification performance in comparison with the clinicians’ interpretations.

	Evaluation Metrics	MLP	SVM	RF	Bagging
**Performance Measure of the Classifiers**	Combined Performance Measure	×	√	√	×
	Model Performance Measure	×	√	√	×
	AUC-ROC	√	√	√	√
	Sensitivity and Specificity	×	√	√	×
	Accuracy of Detecting Suspect Class	√	√	√	×
**Performance Comparison with Clinicians’ Annotation**	Covariance Correlation	√	√	√	×
	Limits of Agreement of B-A Plot	×	√	√	×

## Data Availability

The authors used an open access dataset that is available from https://physionet.org/content/ctu-uhb-ctgdb/1.0.0/.
